# NPD1 Relieves Neuropathic Pain and Accelerates the Recovery of Motor Function After Peripheral Nerve Injury

**DOI:** 10.1155/2024/1109287

**Published:** 2024-10-30

**Authors:** Yu Tian, YanFang Liu, Chang Liu, SaiSai Huang

**Affiliations:** ^1^Department of Anesthesiology, Affiliated Hospital of Nantong University, Nantong, Jiangsu 226001, China; ^2^Medical School of Nantong University, Nantong, Jiangsu 226001, China

## Abstract

The incidence of peripheral nerve injury (PNI) in China is continuously increasing. With an inability to function due to sensory and motor abnormalities, patients with PNI suffer from neuropathic pain and subsequent lesions. Presently, effective treatments for PNI are limited. To determine the role of neuroprotectin D1 (NPD1) in PNI, a sciatic nerve crush injury model was developed to investigate the impact of NPD1 on sensory and motor function recovery following nerve injury. The results demonstrated that NPD1 administered at different time points might reduce mechanical allodynia and thermal hyperalgesia caused by PNI, and its analgesic effect was not tolerated. Immunohistochemistry analyses revealed that administering NPD1 to PNI mice decreased the spinal microglia and astrocyte activation and decreased the inflammatory factor expression in the spinal dorsal horn. Furthermore, NPD1 can inhibit the invasion of IBA-1^+^ macrophages in dorsal root ganglions generated by nerve injury. Meanwhile, it can help rehabilitate motor and neuromuscular functions following PNI. The results indicate that NPD1 may be involved in the sensory and motor function recovery following PNI.

## 1. Introduction

The World Health Organization reports approximately a million new cases of peripheral nerve injury (PNI) worldwide annually [1]. Following PNI, functional recovery is clinically challenging. Neuropathic pain and sensory and motor impairments restrict PNI patients' functions [2–4]. Nevertheless, only a small number of broad-spectrum analgesics have been used as pharmacological therapies for PNI, which achieve unsatisfactory outcomes due to their poor specificity and efficacy for intractable neuropathic pain and many side effects. Thus, new cytokines or pharmacological molecules should be crucially and urgently discovered to suppress neuropathic pain and encourage motor function restoration.

For the treatment of chronic pain, specialized prodecomposing mediators (SPMs) are thought to be a developing analgesic [5–8]. NPD1 is a recently identified member of specialized proresolving mediators. When cell life is in jeopardy, docosahexaenoic acid in the brain produces this lipid messenger on demand [9]. In brain damage studies, this naturally occurring neuroprotective chemical demonstrates potent neuroprotective effects [10–12]. Further studies have demonstrated the effectiveness of NPD1 for the treatment of respiratory tract inflammation, colitis, diabetes-related issues, and inflammatory pain inhibition [13, 14]. These studies verified that NPD1 is involved in reducing inflammation and inflammatory pain. These findings prompted the investigation of whether NPD1 can simultaneously exert anti-inflammatory and analgesic effects and accelerate nerve regeneration following PNI.

This study aimed to examine whether NPD1 could promote nerve regeneration and neurological functional recovery and alleviate neuropathic pain after nerve injury.

## 2. Materials and Methods

### 2.1. Animals

Eight-week-old male C57BL/6 mice were acquired from the Experimental Animal Center of Nantong University. They had free access to water and food and were kept in a 2 h light/dark cycle with a humidity of 55%–60% and a temperature of 23 ± 1°C.

### 2.2. Sciatic Nerve Crush Surgery

All mice were operated under isoflurane inhalation anesthesia with 3%-4% induction and 1%-2% maintenance. The left thigh skin of the mice was cut, and the sciatic nerve was exposed by opening the fascial plane that runs between the anterior head of the biceps femoris and gluteus maximus. A needle holder was used to crush the sciatic nerve for 20 s.

### 2.3. Drugs and Experimental Design

The mice were divided randomly into four groups (*n* = 6). Mice in the naïve group received no treatment; the crush injury model group received the previously mentioned treatment; and the sham group underwent the surgery previously described but did not experience nerve crush. To simulate the effects of short-term prophylactic medication on postoperative neuropathic pain in clinical practice and refer to relevant literature [15], the crush injury model + NPD1 group received 600 ng of NPD1 through the tail vein of the mice at 3, 24, 48, and 72 h after establishing the model. On the hind paw's surgical side, a diluted fluorescent dye (0.5 Vybrant's *μ*l CM-Dil in 20 phosphate-buffered saline [PBS]'s *μ*l) was occasionally administered 7 days following nerve injury. The L5 DRG and L4–L6 spinal dorsal horn sections were examined 7 days following sciatic nerve injury. All mice were sacrificed by intraperitoneally injecting 3% sodium pentobarbital (100 mg/kg), and the spinal dorsal horn and DRG were harvested on day 7.

### 2.4. Behavioral Testing

Using von Frey hairs (0.16–2.0 g, Stoelting Company), mechanical allodynia caused by PNI was evaluated, and Dixon's up–down method was used to compute the withdrawal threshold. Using the Hargreaves device (IITC Life Science, Inc.), heat hyperalgesia was examined. To prevent tissue injury, the radiant heat intensity was set to maintain the basal withdrawal threshold between 10 and 14 s, with a 20-s cutoff.

### 2.5. Quantitative Real-Time PCR

TRIzol reagent from ThermoFisher Scientific, Inc. (cat. no. 15596 026; Invitrogen) was used to purify the total RNA from the spinal dorsal horn. A RevertAid RT Reverse Transcription kit (cat. no. K1691; ThermoFisher Scientific, Inc.) was used to create cDNA. The MiniOpticon Real-Time PCR equipment (Bio-Rad) was used with the 2^−ΔΔCT^ technique for gene-specific mRNA studies to determine the mRNA expression levels.

### 2.6. Immunohistochemistry and Quantification

Isoflurane was used to induce terminal anesthesia in animals, and PBS was then infused into the ascending aorta with 4% paraformaldehyde and 1.5% picric acid. After the extraction, L5 DRGs and L4–L6 spinal cord segments were postfixed in the same fixative for an additional night. In a cryostat, DRGs (10 *μ*m) and the L4–L6 spinal cord (30 *μ*m) were severed. After blocking the DRG sections for 1 h at room temperature with 2% goat or donkey serum, they were incubated with anti-IBA1 (rabbit; 1:800; FuJiFilM Wako Pure Chemical Corporation) primary antibodies for an additional hour at 4°C. Finally, Cy3-conjugated secondary antibodies (1:1,000, Jackson ImmunoResearch Laboratories, Inc.) were used for incubation. The following primary antibodies were used to incubate the spinal cord sections: anti-GFAP (mouse; 1:5000, EMD Millipore) and anti-IBA1 (rabbit; 1:800, FUJIFILM Wako Pure Chemical Corporation). The spinal cord sections were first blocked with 2% goat serum at room temperature for 1 h. The following secondary antibodies were then applied to the sections and incubated for 2 h at room temperature: Cy3 or FITC-conjugated secondary antibodies (1:1000; Jackson ImmunoResearch Labs, Inc.).

### 2.7. CatWalk Gait Analysis

The mouse was left to wander freely on a straight walkway in a pitch-black setting. The CatWalk software automatically recorded their activities as they were moving along a straight path in a dimly lit area. The Noldus CatWalk XT Automated Gait Analysis system was used for data evaluation.

### 2.8. Sciatic Functional Index (SFI) Evaluation

The footprints were obtained by allowing the mice to walk along a 6 × 44 cm corridor on a blank sheet of paper. The SFI was calculated based on the reported methods [16].

### 2.9. Rotarod Test

The rotarod is adjusted to start at 4 rpm and accelerate at a rate of 20 rpm per min. In the test, the mouse was placed on a rotating rod, and as the rod rotated, the mouse needed to constantly adjust its position to maintain balance and avoid falling off. Before they fall for the first time, the duration that they maintain can remain on the rotating pole.

### 2.10. Statistical Analyses

All data were expressed as mean ± standard error of the mean. Behavioral data were analyzed using Student's *t*-test or two-way analysis of variance (ANOVA) followed by Bonferroni post hoc test. Electrophysiology data were analyzed using paired or unpaired Student's *t*-test. LTP data were tested using two-way ANOVA. The criterion for statistical significance was *p* < 0.05.

## 3. Results

### 3.1. Sciatic Nerve Crush Injury Induces Motor Dysfunction and Neuropathic Pain in Mice

**Figure 1:**
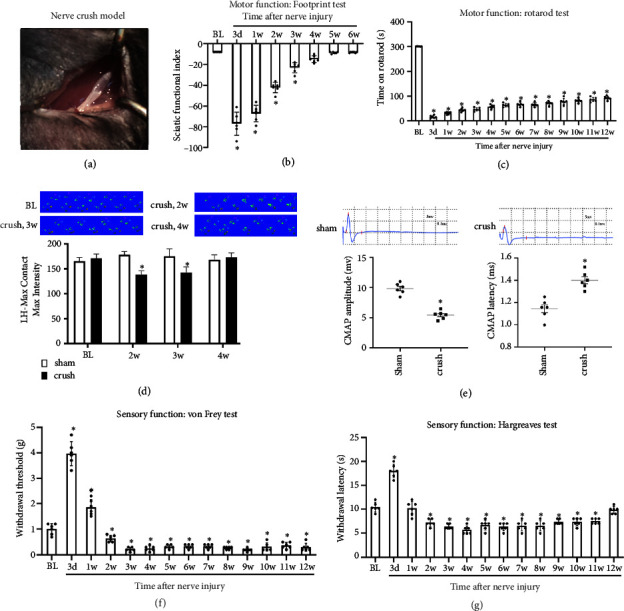
(a) Sciatic nerve crush injury model was established. The motor functions of mice were measured by SFI values (b), rotarod tests (c), and Catwalk gait analysis (d). The neuromuscular function postnerve injury was measured using CMAP (e). The sensory functions of mice were measured using the von Frey (f) and Hargreaves (g) tests. ⁣^∗^*p* < 0.05 vs. the baseline (BL) group and ⁣^∗^*p* < 0.05 vs. the sham group. BL, baseline before nerve injury. Data are presented as the mean ± SD, *n* = 6 mice in each group.

To evaluate the motor function recovery and allodynia reduction following nerve injury, a sciatic nerve injury of mice was created. Catwalk gait analysis and SFI value detection were used to assess the basic motor function recovery following nerve injury. The findings indicate that walking—the most fundamental motor function—did not completely recuperate for 4 weeks (Figures 1(b), 1(d)). The rotarod test was used to examine more closely at how fine motor function recovers following nerve injury. The findings revealed that in comparison to normal mice, nerve-damaged animals remain on the revolving rod for a considerably shorter period before their initial fall (⁣^∗^*p* < 0.05) (Figure 1(c)). Furthermore, compound muscle action potentials (CMAPs) recording was used to evaluate the motor function recovery. Figure 1(e) illustrates how, at 3 weeks following sciatic nerve crush, the injured side's CMAP amplitude was smaller, and its latency was longer than that of the sham group (⁣^∗^*p* < 0.05) (Figure 1(e)). These results demonstrated that after PNI, basic and fine motor functions of mice are affected.

After the nerve injury, thermal hyperalgesia and mechanical allodynia were assessed by testing mechanical and thermal pain thresholds. After the nerve damage, a shift from hypoesthesia to hyperalgesia was observed. Significant hyperalgesia was detected in the afflicted limb 2 weeks after modeling and persisted for at least 11 weeks (Figures 1(f), 1(g)).

### 3.2. NPD1 can Prevent and Reverse Neuropathic Pain Without Signs of Antinociceptive Tolerance

**Figure 2:**
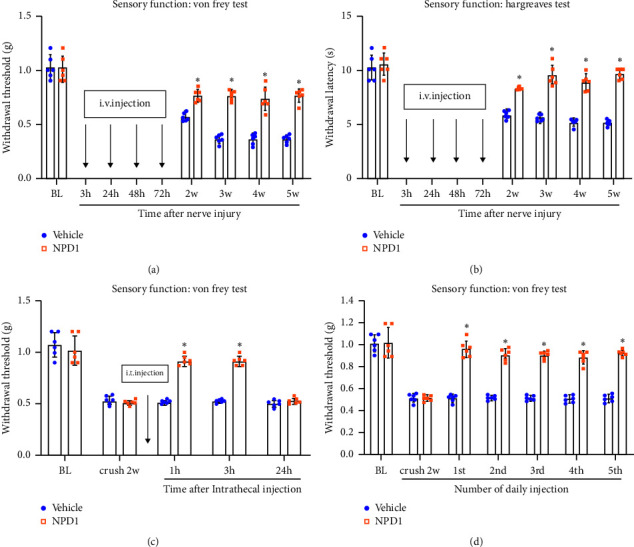
NPD1 prevented and reversed neuropathic pain without tolerance. (a and b) NPD1 (600 ng, caudal vein) prevented mechanical and thermal hyperalgesia in mice at 3, 24, 48, and 72 h after PNI. (c) At 2 weeks post-PNI, intrathecal injection of NPD (100 ng) reduced mechanical hyperalgesia. (d) Repeated intrathecal injections of NPD1 (once per day for 5 days) produced sustained PNI-induced mechanical hyperalgesia reduction, tested 1 h after each injection. ⁣^∗^*p* < 0.05 vs. vehicle group. Data are presented as the mean ± SD, with 6 mice for each group.

At 3, 24, 48, and 72 h after PNI, mice were injected with NPD1 600 ng via the tail vein. The thermal and mechanical allodynia–hyperalgesia of the surgical side was then assessed. The findings demonstrated that NPD1 might decrease nerve injury-related thermal and mechanical hyperalgesia compared with the vehicle group (⁣^∗^*p* < 0.05) (Figure 2(a) and 2(b)), indicating that NPD1 intervention can successfully stop the neuropathic pain development at the early stages after PNI.

In this study, the possibility that NPD1 reduces chronic neuropathic pain was also evaluated. In PNI mice, neuropathic pain had developed after 2 weeks of modeling. Intrathecal injection of 100 ng NPD1 reversed mechanical hyperalgesia in less than an hour and continued for over 3 h (Figure 2(c)), demonstrating that NPD1 can reverse the already formed neuropathic pain symptoms. After 2 weeks of modeling, the sheath was injected with 100 ng of NPD1 once a day for 5 days in a row. The findings revealed that administering NPD1 2 weeks after modeling still provided pain relief without showing apparent signs of antinociceptive tolerance (Figure 2(d)).

**Figure 3:**
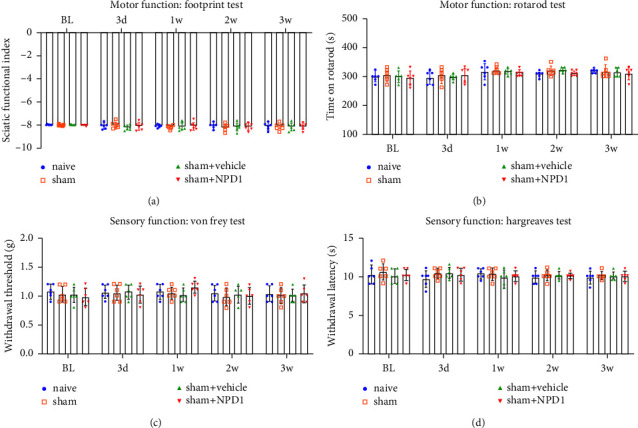
NPD1 had no adverse influence on the sensory and motor abilities of normal mice. Motor functions of PNI mice were measured using footprint (a) and rotarod (b) tests. The sensory functions of PNI mice were measured using the von Frey (c) and Hargreaves (d) tests. Data are presented as the mean ± SD, with 6 mice for each group.

To determine whether NPD1 had an adverse influence on the sensory and motor abilities of normal mice, we ran a second 3-week trial. Postoperatively, mice in the sham surgery group (surgeons performing surgery without compressing the sciatic nerve) were given either NPD1 or a vehicle. The findings demonstrated that NPD1 did not induce mechanical or thermal hypersensitivity in the mice, compared with the sham group or the sham + vehicle group (*p* > 0.05); additionally, NPD1 did not adversely affect the mice's behavior or motor performance, compared with sham group or sham + vehicle group (*p* > 0.05) (Figure 3). These results indicate that NPD1 does not have adverse effects on the sensory and motor functions of normal mice.

### 3.3. NPD1 Inhibited Inflammation and Neuroglia Cell Activation in the Spinal Cord After PNI

**Figure 4:**
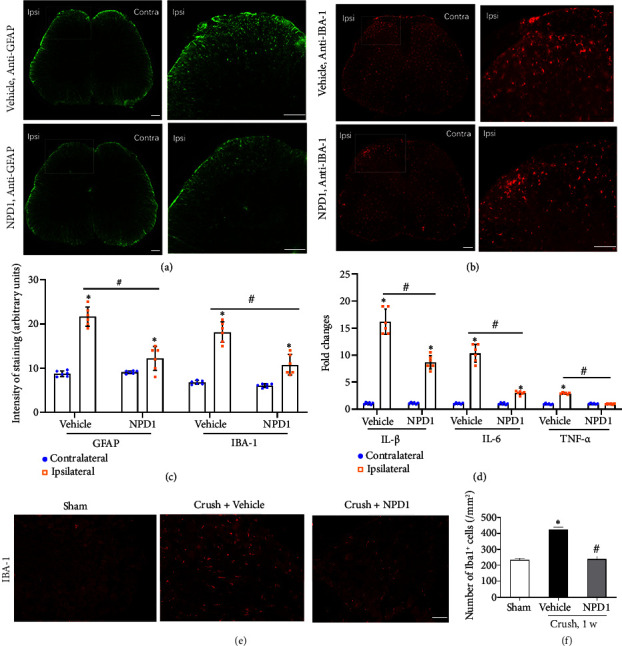
(a–c) NPD1 suppressed the astrocyte and microglia activation in the spinal dorsal horn 7 days after PNI. Scale bar = 50 *μ*m. (d) NPD1 inhibited the increased Il-1*β*, Il-6, and TNF-*α* expressions in the spinal dorsal horn induced by PNI 7 days posttreatment. (e and f) NPD1 prevented PNI-induced infiltration of IBA-1^+^ macrophages in DRGs 7 days posttreatment. Scale bar = 50 *μ*m. ⁣^∗^*p* < 0.05 vs. the contralateral group; ^#^*p* < 0.05 vs. the vehicle group. Data are presented as the mean ± SD, with six mice in each group.

Following PNI, continual nociceptive impulses are sent to the spinal dorsal horn via the DRG. Previous studies have reported that preserving neuroglia cells in their resting state are essential for the dynamic equilibrium of the central nervous system. Double immunohistochemistry analysis was used to assess the expression of the microglial marker IBA-1 and astrocytic marker GFAP in L4–L6 spinal dorsal horn 7 days after PNI to better determine the potential mechanism of NPD1 in inhibiting neuropathic pain. The findings demonstrated that the immune response intensity of IBA-1 and GFAP was significantly higher in the spinal dorsal horn of the model group of mice 7 days after PNI in comparison to the contralateral side (⁣^∗^*p* < 0.05) (Figures 4(a), 4(b), and 4(c)). Compared with the vehicle group, PNI mice demonstrated a significant decrease in the immunological response intensity of GFAP and IBA-1 in the ipsilateral spinal dorsal horn following the NPD1 administration (^#^*p* < 0.05) (Figures 4(a), 4(b), and 4(c)). This finding indicates that NPD1 can considerably reduce the activation of neuroglia cells in the ipsilateral spinal cord in mice with PNI-induced neurological injury.

Inflammatory factors are critical for neuropathic pain occurrence and persistence. The IL-1*β*, IL-6, and TNF-*ɑ* expressions in the ipsilateral spinal dorsal horn were higher than those on the contralateral side 7 days after PNI (⁣^∗^*p* < 0.05). Nevertheless, the IL-1*β*, IL-6, and TNF-*ɑ* expressions were significantly lower in the NPD1 group than in the vehicle group (^#^*p* < 0.05) (Figure 4(d)).

### 3.4. NPD1 Prevents PNI-Induced Injury Responses in DRGs

Cell bodies of primary sensory neurons are located in the DRG, providing peripheral nociceptive sensation to the spinal dorsal horn. A study [17] reported that following nerve injury, the wounded nerve, DRG, and spinal dorsal horn can all show signs of inflammatory cell infiltration and immune cell activation. The macrophage counts significantly increase in DRG after PNI and development of mechanical hypersensitivity reactions brought on by PNI can be markedly suppressed by macrophage depletion in DRG [17, 18]. After modeling, mice were administered 600 ng of NPD1 via the tail vein at 3, 24, 48, and 72 h. At 1 week after modeling, L4–L6 DRGs on the same side of nerve injury were taken. IBA-1 immunostaining reveals that the number of IBA-1^+^ macrophages in DRGs was significantly higher in the vehicle group than in the sham group (⁣^∗^*p* < 0.05); a significant decrease of the number of IBA-1^+^ macrophages was also observed in DRGs in the NPD1 group compared with the vehicle group (^#^*p* < 0.05). The results demonstrated that NPD1 prevents PNI-induced infiltration of IBA-1^+^ macrophages in DRGs, protecting DRG neurons from immune damage and further preventing pain signals from being transmitted to the central nervous system (Figure 4(e)).

### 3.5. NPD1 Promoted the Motor and Neuromuscular Function Recovery After PNI

**Figure 5:**
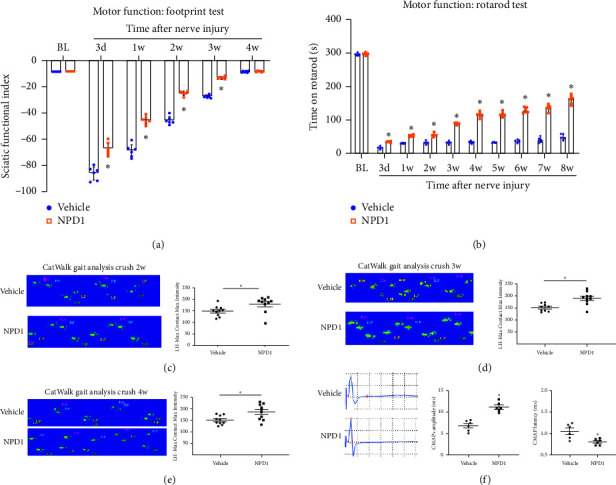
(a and b) Footprint and rotarod tests in PNI mice treated with NPD1. (c–e) Catwalk gait analysis at 2, 3, and 4 weeks after PNI in mice treated with NPD1. (f) CMAP recordings in the PNI mice treated with NPD1. ⁣^∗^*p* < 0.05 vs. the vehicle group. Data are presented as the mean ± SD, with six mice in each group.

Using the catwalk gait analysis, rotarod test, and CMAP, motor performance and muscle electrical activity were assessed to determine whether NPD1 can support functional recovery following nerve injury. The afflicted hindlimbs' SFI values in the NPD1 group were significantly higher than those in the vehicle group (⁣^∗^*p* < 0.05) (Figure 5(a)). Using the rotarod test, the fine motor function of PNI mice was evaluated. Compared with the vehicle group, mice treated with NPD1 remained on the revolving rod for a noticeably longer period before the first fall (⁣^∗^*p* < 0.05) (Figure 5(b)). Furthermore, in PNI mice, NPD1 markedly increased the max contact intensity of the impacted hindlimbs compared with the vehicle group (⁣^∗^*p* < 0.05) (Figures 5(c), 5(d), and 5(e)). These findings indicate that NPD1 treatment reduced the basic and fine motor dysfunctions brought on by nerve damage in the affected limbs. Following a sciatic nerve crush injury, CMAP of the hindlimb muscles was recorded. The results indicate a significant increase of the amplitude of CMAP and a decrease of its latency in the NPD1 group compared with the vehicle group (⁣^∗^*p* < 0.05), demonstrating neuromuscular function recovery (Figure 5(f)).

## 4. Discussion

PNI is a serious clinical and public health issue frequently resulting in substantial functional impairment and lifelong disability [19]. Sensory and motor impairments prevent patients with PNI from doing activities, and they also experience neuropathic pain and its associated lesions. This study focused on how nerve function recovers following PNI.

Sciatic nerve crush injury model, the most widely used PNI model in rodents, was used [20, 21]. All axons are broken by the nerve crush in this model; however, the basal lamina and Schwann cells are left intact, allowing regeneration in 3 to 4 weeks [22]. A new sciatic nerve crush model was developed for this study, and the model mice underwent a series of behavioral tests to evaluate both neuropathic pain and motor function. Mechanical allodynia testing, catwalk gait analysis, CMAP measurements in the gastrocnemius muscle, and SFI evaluation were conducted to track the degree of neuronal degradation and regeneration over time. Our results revealed that the model showed steady recovery curves (Figure 1), indicating that it is a good model to evaluate the neuropathic pain and motor function recovery. This study aims at assessing the effects of NPD1 on the neuropathic pain and motor function recovery following PNI.

NPD1 is recognized as a recently identified SPM member. It has been reported to be very beneficial for the treatment of colitis, respiratory tract inflammation, issues from diabetes, and inflammatory pain inhibition. This study found that NPD1 intervention can successfully stop the neuropathic pain development at the early stages after PNI. Early intervention with NPD1 in patients may reduce the incidence of postoperative neuropathic pain in clinical practice. For neuropathic pain that has already developed in the later stages of major surgery in clinical practice, no effective treatment option is currently available. Postoperatively, patients continue to feel excruciating agony for weeks, months, or even years. Our study findings indicate that NPD1 can reverse neuropathic pain without signs of antinociceptive tolerance.

Nerve growth factor (NGF) is one of the most researched growth factors now used for the treatment of PNI. Neuronal development and survival are significantly influenced by NGF [23–25]. Nevertheless, the adverse effect of NGF-producing neuropathic pain has not received much attention from researchers investigating its impact on PNI [26–28]. The paw withdrawal threshold and latency were decreased after injecting NGF into the plantar tissues of normal mice [29]. In this study, mice injected with NPD1 showed no changes in these parameters while relieving neuropathic pain. Furthermore, NPD1 did not negatively affect the mice's behavior in motor function (Figure 2). The results indicate that NPD1 may have an advantage over NGF in treating neuropathic pain.

Following PNI, the wounded nerve, DRG, and spinal dorsal horn can all show signs of inflammatory cell infiltration and immune cell activation. Our crush injury model for PNI significantly increased the number of macrophages in the DRG on the side of nerve injury. Remarkably, NPD1 can prevent the invasion of Iba1^+^ macrophages in DRGs, thus protecting DRG neurons from immune damage and further inhibiting the transfer of pain signals from the DRG to the central nervous system.

Neuropathic pain pathophysiology is significantly influenced by neuroinflammation, a localized inflammation within the nervous systems [29–31]. Activated neuroglia cells and increased inflammatory factor expression are the main characteristics of the nerve injury-induced neuroinflammatory response [32]. In the current work, mice with PNI developed mechanical allodynia and thermal hyperalgesia as well as a substantial increase in activated microglia and astrocytes in the spinal cord. Concurrently, PNI increased the IL-1*β*, IL-6, and TNF-*α* expressions. Neuroglia cell activation and increased inflammatory factor levels in the spinal cord were dramatically reduced by systemic administration of NPD1 in PNI mice. These findings indicate that NPD1 modulates glial activity and inflammatory factor expression to reduce neuropathic pain caused by PNI.

This study also described the motor function recovery following PNI. Starting 4 weeks following the injury, no discernible difference was observed between the fundamental motor and normal functions in PNI mice. However, even at 12 weeks after establishing the model, the fine motor function of PNI mice was not entirely restored. When NPD1 was administered for 3 h, 24 h, 48 h, and 72 h following nerve damage, the basic and fine motor dysfunctions of the limbs were reduced. CMAP recordings can be used to track the degree of motor neuron degeneration and regeneration over time [33]. NPD1 can extend the latency of CMAP and decrease its amplitude in PNI mice. These findings indicated that NPD1 treatment was beneficial for motor function and neurotransmission repair following sciatic nerve damage.

In summary, these results demonstrate that NPD1 effectively prevents and reduces neuropathic pain caused by PNI mechanistically by [1] protecting DRG neurons from immune damage and [2] preventing nerve injury-induced spinal glial reaction and proinflammatory responses. Furthermore, NPD1 can improve the motor function recovery after PNI, but its mechanism still needs further exploration.

This study has inherent limitations that should be considered. First, its relatively small sample size may limit the generalizability of the findings. Second, due to the research conditions, this experiment did not compare the effects of NGF and NPD1 on both the sensory and motor functions of PNI mice which will be further improved in future studies.

## 5. Conclusions

The findings of this study supplement previously published information demonstrating the antinociceptive effects of NPD1 therapy on various PNI models. NPD1 not only treated neuropathic pain after PNI but also accelerated the recovery of damaged nerve motor function. Therefore, NPD1 may be considered a novel analgesic agent for the treatment of neuropathic pain and as a new activator for nerve regeneration. However, further research is warranted to verify our findings and identify plausible underlying mechanisms.

## Data Availability

The data that support the findings of this study are available from the corresponding author upon reasonable request.
